# Ethnobotanical Study of Medicinal Plants Used to Treat Human Diseases in Berbere District, Bale Zone of Oromia Regional State, South East Ethiopia

**DOI:** 10.1155/2018/8602945

**Published:** 2018-07-17

**Authors:** Tilahun Tolossa Jima, Moa Megersa

**Affiliations:** ^1^Department of Biology, Ambo University, P.O. Box 19, Ambo, Ethiopia; ^2^Department of Environmental Health Science and Technology, Jimma University, P.O. Box 378, Jimma, Ethiopia; ^3^Department of Biology, Madawalabu University, P.O. Box 247, Robe, Ethiopia

## Abstract

This paper reports an ethnobotanical study that focused on the traditional medicinal plants used by local communities to treat human diseases. Ethnobotanical study of medicinal plants was carried out from June 25 to September 5, 2015, in Berbere district of Oromia region, Ethiopia. The study focused on documentation of medicinal plants used to treat various human diseases in the study area. Ethnobotanical data were collected using semistructured interviews, group discussion, guided field walks, and observations with participants. Preference ranking, paired comparison, direct matrix ranking, and informant consensus factors (ICF) were used to analyze the importance of some plant species. A total of 70 medicinal plants, distributed in 56 genera and 46 families, were collected and identified. Plant family with the highest medicinal plants in the study area used for various diseases treatment was Euphorbiaceae (11.4%). The result of growth form analysis showed that shrubs constituted the highest proportion of medicinal plants (48.6%). Roots, 43 (44.8%), were the most frequently utilized plant parts for preparation of traditional herbal medicines. Crushing was a widely used mode of preparation of traditional remedies where oral administration (37.5%) was the dominant route. The highest informants consensus factor (ICF) values were linked to gonorrhea and syphilis disease (0.95); the lowest was linked with external parasites and wound (0.69). Local people in the study area possess traditional knowledge of medicinal plants to treat various human ailments; however, agricultural expansion and disinterest of young generation became the major threat to medicinal plants. It is, therefore, necessary to preserve this indigenous knowledge on traditional medicines by proper documentation, identification of plant species used, and herbal preparation. To save medicinal plants from further loss, involving local communities in cultivation of the most utilized medicinal plants is recommended.

## 1. Introduction

Plant resources have remained an integral part of human society throughout history. After fulfilling the primary needs like food and shelter, man has sought for a suitable remedy among plants for curing various diseases [1].

Traditional medicine is defined as indigenous medicine that is used to maintain health and to prevent, diagnose, and treat physical and mental illnesses differently from allopathic medicine based on theories, beliefs, and experiences [2]. Traditional medicine has been used for thousands of years with great contributions made by practitioners to human health, particularly as primary health care providers at the community level and has maintained its popularity worldwide [3]. According to Sofowora [4], about 60-85% of the population in every country of the developing world has to rely on traditional medicine. The practice of traditional medicine is widespread in China, India, Japan, Pakistan, Sri Lanka, Thailand, and Korea [5]. In China, traditional medicine accounts for around 40% of all health care delivered and is used to treat roughly 200 million patients annually [6].

In Ethiopia, plants have been used as a source of medicine from time immemorial to treat different ailments due to its long history, and traditional medicine has in fact become an integral part of culture [7]. These traditional medical practices and remedies are recorded in oral tradition and in early medico-religious manuscripts and traditional pharmacopoeias, which, according to the estimates of some historians, date back to the 15th century AD [8].

Ethiopia possesses about 6,000 species of vascular plants which could be due to its different topography and climatic conditions [9]. About 80% of human population and 90% of livestock rely on traditional medicine in this country [10]. Traditional medicine of Ethiopia is commonly used to treat various human and livestock ailments. Traditional healers known by different names in different parts of the country are the primary players in the curative aspect of traditional medicine practice [11]. Thus, this study was initiated to document the traditional medicinal plants knowledge accumulated by local communities of Berbere district.

## 2. Methods

### 2.1. Study Area

**Figure 1:**
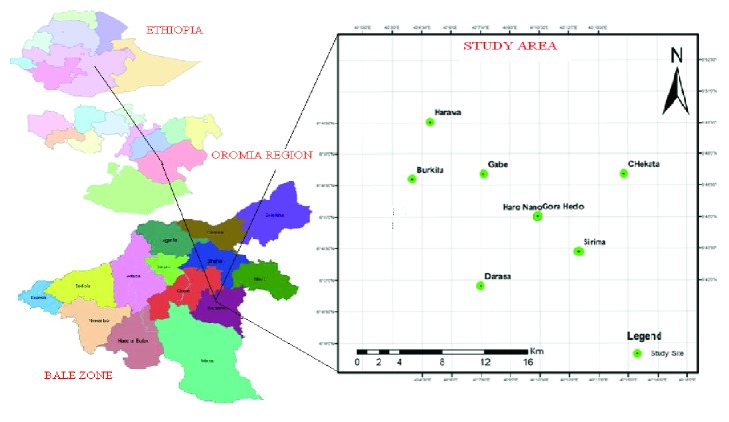
Map showing study area.

Berbere district is situated between 06°33′ N and 06°75′N and 039°95′ E and 040°29′E. It is located at about 526 km southeast of Addis Ababa, in Bale Zone of Oromia Regional State. This district has 17 kebeles which are characterized by undulating highlands in the north and lowlands in the south (Figure 1).

### 2.2. Selection of Study Sites

A reconnaissance survey of the study area was conducted from June 25 to July 5, 2015. The study sites were selected depending on recommendation from elders, local authorities, and altitudinal range. Thus, the study was carried out in eight kebeles from two agro-climatic zones.

### 2.3. Selection of Participants

A total of 60 participants (41 men and 19 women) were selected randomly from the representative kebeles. Representative common participants and knowledgeable traditional medicine practitioners (key participants) of Berbere district were selected using random and purposive sampling approaches, respectively, following Martin [12]. Twenty key participants were selected purposively and systematically based on the recommendations of knowledgeable elders, local authorities, and development agents. The selection of key participants was also based on the quality of explanations that particular participants gave during an interview. Local healers automatically qualified as key participants being traditional experts who are guardians of indigenous knowledge on medicinal plants.

### 2.4. Ethnobotanical Data Collection

Ethnobotanical data were collected from July 6, 2015, to September 5, 2015. The standard data collection methods [12–14] have been followed to document indigenous knowledge of the local community on health, use, conservation, and threats of medicinal plants. The techniques employed for data collection were semistructured interviews, group discussion, guided field walks, and observations with participants. Semistructured interviews were undertaken based on checklist of questions prepared in English and translated to ‘Afaan Oromo', the language of local people. Information was carefully recorded during an interview with a participant. The interview was guided to cover the key topics on the checklist. The place and the time for interview were set based on the interest of the participants. Field observations were performed with the help of local guides on the morphological features and habitats of each medicinal plant species in the field. Brief group discussions were made with participants regarding the medicinal plants in the study area. The discussions were conducted on threats to medicinal plants, conservation of the medicinal plants, and transferability of knowledge in the community. Letter of consent was taken from Jimma University, prior to the data collections. Verbal consents were also obtained from the participants by performed group discussions about the objectives of the study prior to the interviews, and all data were collected through their oral consents.

### 2.5. Voucher Specimen Collection

The voucher specimens were collected onsite during guided field walk, numbered, pressed, dried, and deep frozen for identification. Identification of specimens was carried out both in the field and in the herbarium. Identification was also carried out using Flora of Ethiopia comparing with already identified specimens. Finally, the identified specimens were stored at the National Herbarium of the Addis Ababa University, Ethiopia.

### 2.6. Data Analysis

The collected ethnobotanical data were entered into Excel spreadsheet 2007 and summarized using descriptive statistical methods such as frequency, percentage, tables, and graphs. Preference ranking and paired comparison were computed following [13]. Preference ranking was conducted for five important medicinal plants used to treat stomachache. Ten randomly selected participants from total key participants were participated in this exercise to identify the best preferred medicinal plants for treatment of stomachache.

In paired comparison, ten participants were selected and asked to choose the best item from every pair according to personal perception in treating wound. The total number of possible pairs [15] was obtained by applying the formula** n (n-1)/2**, where** n **is the number of medicinal plants being compared. A total rank of paired comparison was obtained by summing the number of times each item was chosen. An item with the highest frequency of choices had the highest score.

Direct matrix ranking is used to compare multipurpose uses of a given plant species based on information gathered from participants, number of multipurpose species were selected out of the total medicinal plants, and use diversities of these plants were listed for four randomly selected key participants to assign use values to each species.

Informants consensus factor (ICF) was calculated for each category to identify the agreements of the participants on reported cures for the group of ailments. The ICF was calculated as follows [15].

(1) $$\begin{array}{c} ICF=\frac{nur-nt}{nur-1} \end{array}$$

where ICF is informants consensus factor, nur is number of use citation in each category, and nt is number of species used.

## 3. Results and Discussion

### 3.1. Medicinal Plants of the Study Area

A total of 70 medicinal plant species belonging to 56 genera and 39 families were used by the local communities to treat 42 human ailments (Table 1). Euphorbiaceae was the leading family with eight species (11.4%). Of the ethnomedicinally important plant species that are used to treat human ailments recorded in eight kebeles of Berbere district, 12 (19%) were from homegardens and 58 (81%) species were from the wild. Various studies conducted in Ethiopia reported that most of medicinal plants are being harvested from noncultivated areas. For instance, the study conducted by [16] indicated that the highest number (90.43%) of medicinal plants was collected from wild in Mana Angetu District. Similarly, [17, 18] reported that about 54% and 49% of medicinal plants were harvested from wild in Tehuledere and Halaba districts, respectively. This observation is a good indication of the fact that the local people have not yet started cultivating most of the plant species they are using as remedies.

### 3.2. Growth Form of Medicinal Plants

**Figure 2:**
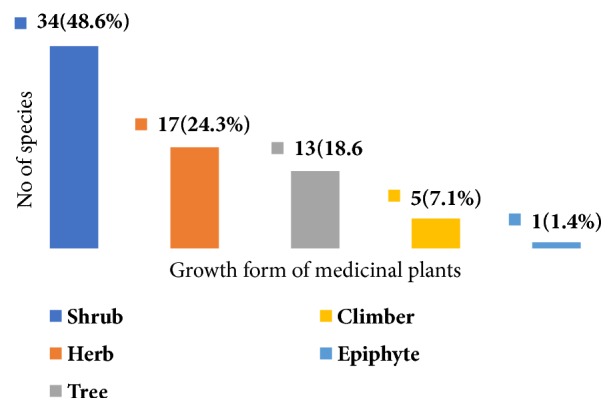
The habits (growth forms) of medicinal plants used to treat human ailments in the study area.

The growth form analysis of medicinal plants revealed that shrubs constitute the highest proportion being represented by 34 species (48.6%), herbs represented by 17 species (24.3%), trees represented by 13 species (18.6%), and climbers represented by 5 species (7.1%), while there was one species (1.4%) of epiphyte (Figure 2).

This finding shows that the most represented life forms of medicinal plants in the study area were shrubs followed by herbs. Similar findings were also reported in the earlier works in Ethiopia. For instance, [19] identified 46.8% shrubs and 24.1% herbs from Cheffa semi-wetland; [17] documented 46% shrubs and 25% herbs from Boosat subdistrict; [20] collected 37% of shrubs from Sekoru district, Jimma Zone. The more recent studies conducted by [21, 22] also reported the dominance of shrubs for preparation of traditional medicines. However, other findings [18, 23–25] indicated that herbs were the most frequently used plant categories.

### 3.3. Plant Parts Used to Treat Human Diseases

**Figure 3:**
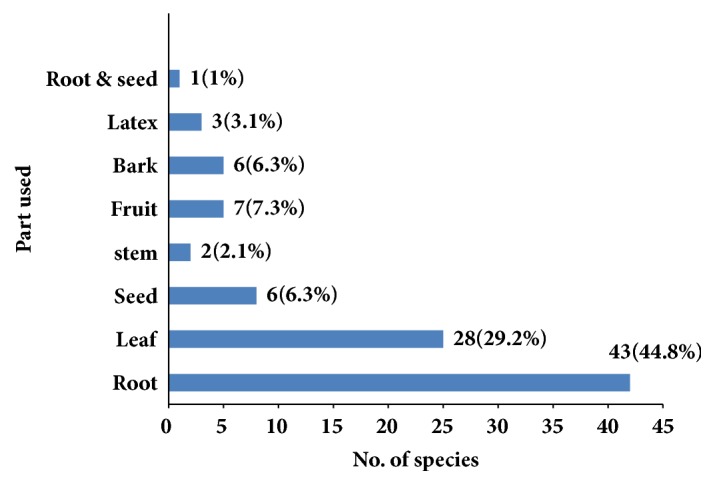
Plant parts used in human traditional medicine.

People of the study area harvest different plant parts for the preparation of traditional remedies (e.g., leaves, roots, seeds, barks, and fruit). In the study area, 43 species (44.8%) were harvested for their roots (Figure 3). This is because it is believed that roots contain more concentration of the active ingredients.

The finding of the roots as the contributor of higher number of plant species used for medicinal purpose than other plants parts is in line with similar study conducted by [17], in which roots (31.4%) were reported as the most widely used plant part followed by leaves 24.4%. The study conducted by [20] showed that roots 42% as a major plant parts used and [26] also documented root 35.8% as a major plant parts used in the treatment of human ailments. In this regard, [27] have indicated that plant harvest involving roots, rhizomes, bulb, bark, and stem has a serious effect on the survival of the mother plant in its habitat. However, the findings of [18, 21, 28–30] indicated that traditional medicinal preparations mainly involve the use of leaves. As leaves of medicinal plant species were also reported to be harvested for most remedy preparations next to roots, collection of leaves could be promoted as a more sustainable method since in most cases at least many leaves are left over on the parent plant [24].

### 3.4. Method of Preparation, Dosage, and Administration

In the collection of data concerning the preparation of medicine, participants reported various skills associated with herbal preparation. The results showed that the most remedies were prepared from a single plant or plant parts (73%) and preparation from combined plant species was 27%. Similarly, various research findings reported the use of single plant species or parts for traditional remedy preparation [22, 23, 31]. The potency of using a mixture of different plant or plant parts increased compared to using a single plant to cure a disease is evident.

The majority of the remedies (78.6%) in the study area were prepared from fresh parts of medicinal plants followed by dried form (15.7%) and (5.7%) prepared either from dry or fresh plant parts. The study conducted by [32] indicated that 86% of preparations were in fresh form and [33] reported that most of (64%) medicinal plants were used in fresh form and 36% in dried from. The dependency of local people on fresh materials is mostly due to the effectiveness of fresh medicinal plants in treatment as the contents are not lost before use compared to the dried forms.

**Figure 4:**
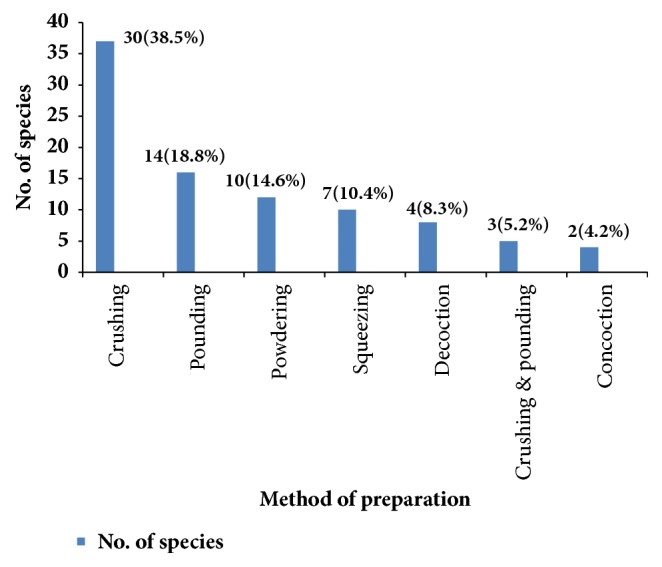
Mode of preparation of human medicinal plants in the study area.

The local communities employ various methods of preparation of traditional medicines for different types of diseases. The preparations vary based on the type and actual site of disease treated. The principal methods of remedy preparation were reported to be through crushing, which accounted for 30 preparations (38.5%) followed by pounding which accounted for 14 preparations (18.8%), powdering accounted for 10 preparations (14.6%), squeezing accounted for 7 preparations (10.4%), decoction accounted for 4 preparations (8.3%), crushing and pounding together accounted for three preparations (5.2%), and 4.2% of traditional medicines were prepared using concoction (Figure 4). Crushing as the most common mode of preparation is in agreement with the findings of [20, 34] who noted that the principal method of remedy preparation was through crushing. However, [27] in a similar study on people of Wonago District reported that powdering was a dominant method of preparation of remedy.

Medicinal plants were applied through different routes of administration (Table 1). In the study area, the substantial proportions of prescriptions were administered orally (37.5%), followed by external application (13.5%), creaming (10.4%), and dry bath (8.3%). This result is in line with the findings of [29, 32, 35]. Internal ailments were commonly treated by making the patient drink herbal preparations; tooth infection was treated by crushing and applies on the remedial plant part on the tooth surface; skin infections such as ringworm were treated by creaming herbal preparations on an infected skin.

Concerning the dosage, local people of the study area used various units of measurement such as finger length (e.g., for root, bark, and stem), pinch (e.g., for powdered), and numbers (e.g., for leaves, seeds, fruits, and flowers) and locally made spoons from plants were used to estimate and fix the amount or dosage of medicine. For human disease treatment, the smallest number is one and the highest is 7 for the case of leaves. In the case of palm measurement units, the lowest is half palm and the highest is 3 palms depending on the age of the patients. The lack of precise dosage is one of the drawbacks of traditional medicinal plants [33, 36]. Tip part of the index figure is commonly used as a measurement of dose [37]. The result of [19] from Tehuledere district, South Wollo, showed that about 31% preparations were taken with known dosages mostly quantified by spoon, cup of tea, palm, and other equipment. The majority plant remedies (69%), however, are taken with no fixed dosage.

According to respondents, preparations were prescribed to patients differently for different age groups, sex, and other conditions. The dosage prescription for children was mostly lower than for adults. The amounts of remedy and prescription rates were generally dependent on the degree and duration of the ailment. Treatment durations varied between 1 and 7 days. This result is in line with the result of [29] in which treatment durations varied between 1 and 7 days.

Recovery from the disease, disappearance of the symptoms of the diseases, fading out of the disease sign, and judgment of the healer to stop the treatment were some of the criteria used in determining duration in the administration of the dosage. Most of the remedies were reported to have no serious adverse effects except vomiting and temporary inflammations. This could be attributed to the low toxicity of the remedy preparations of the medicinal plant species used by the traditional healers in the study area. A similar study by [36] indicated that some herbal preparations are considered harmless.

### 3.5. Knowledge on Medicinal Plants

Ethnomedicinal knowledge is concentrated in the elderly and relative members of the community and difficult in its transfer from the elders to the young generation. Most of the respondents (79%) preferred to transfer their indigenous knowledge to their family verbally and the remaining participants (21%) through showing the medicinal plants in the fields. Indigenous knowledge transfer to the young generation is considered poor which may cause erosion of the practice. The study revealed that medicinal plant knowledge transfer to the young generation is affected by modernization. This might be related to the diminishing of interest of the young generation on indigenous knowledge. Similar result was reported by [20] where young people showed disinterest on traditional medicinal plants.

### 3.6. Ranking of Medicinal Plants

Preference ranking of 5 medicinal plants that were reported as effective for treating stomachache was conducted after selecting ten key participants. Stomachache is the mostly occurring disease treated by more medicinal plants. The participants were asked to compare the given medicinal plants based on their efficacy. The results showed that* Stephania abyssinica* scored the highest mark and ranked first indicating that it was the most effective in treating stomachache and followed by* Solanum incanum *(Table 2).

For medicinal plants that were identified by the participants to be used in treating wounds, a paired comparison was made among six of them using ten participants to know their rank. Wound is frequently occurring external health problem in the study area. The results that were obtained from all the participants were summed up and compared plants were abbreviated in the table. Accordingly,* Olea europaea *subsp.* cuspidata* selected five times and stood first indicating that it is the most effective in treating wound followed by* Prunus africana, Euphorbia heterophylla, Acacia senegal, Cyphostemma *sp*., and Clematis simensis *(Table 3). The frequency of wound to be treated by more medicinal plants was also reported by [38] in other parts of Bale region. Nine medicinal plants were documented for wound treatment.

In the study area, a number of medicinal plants were found to be multipurpose species being utilized for a variety of uses. Direct matrix ranking showed that of the total medicinal plants* Croton macrostachyus* is the most multipurpose use medicinal plant followed by* Warburgia ugandensis *(Table 5). The finding of [39] also reported* Croton macrostachyus *as the highest multipurpose use in Farta district South Gonder Zone of Amhara Regional State.

### 3.7. Informant Consensus Factor (ICF)

The diseases of the study area have been grouped into various categories based on the site of occurrence of the disease, condition of the disease, and treatment resemblance of the disease to the local people. The informant consensus factors have been calculated for each category (Table 4). The highest ICF (0.95) value was obtained for diseases related to gonorrhea and syphilis and the least one (0.69) was associated with boils, dandruff, eczema, haemorrhoids, scabies, Tinea versicolor, and wound.

### 3.8. Threat and Conservation of Medicinal Plants of Study Area

Medicinal plants are at increasing risk from destruction of their habitats (agricultural expansion, fire, construction, overgrazing, and urbanization) and over harvesting of known medicinal species [40]. According to [41] about 15,000 medicinal plant species may be threatened with extinction world widely due to habitat loss and over harvesting and it is estimated that the earth is losing one potential major drug every two years. In the study area, the people also rely on medicinal plants for various purposes such as charcoal, medicine, firewood, construction, and food. The major threat to medicinal plants in the study area was agricultural expansion which accounted for 55%, charcoal production (15%), and fire wood accounted for 18%. The result of [42] indicated that intense deforestation became the major threat to medicinal plants in Zay people. According to [20] deforestation (40%) and agricultural expansion (12.5%) were the major threat to medicinal plants of Sekoru district of Jimma zone. Overgrazing reported to be the major threat to medicinal plants in Gozamin Woreda of East Gojjam [43].

This indicates that due consideration should be given for conservation of these plants since they are being widely exploited for purposes other than their medicinal value. Availability of medicinal plants has been affected by a dramatic decrease in the area of native vegetation due to agricultural expansion, deforestation, fire, overgrazing, and charcoal and firewood [40, 42].

The influences of human on the natural habitat of medicinal plants are the problems for the conservation of medicinal plants and associated knowledge. The effort to conserve medicinal plants in the district was observed to be very poor. Some participants have started to conserve medicinal plants by cultivating at home gardens, though the effort was minimal. About 13.8% of the medicinal plants collected were reported as found cultivated at home gardens and these include plants like* Carica papaya*,* Coffea arabica,* and* Jatropha curcas. *The result of [32] indicated that only 5.7% of medicinal plants were cultivated in home garden showing minimal effort of medicinal plant conservation in Mena Angetu district.

Suggestion given by [44] stated that natural resources could be utilized best in sustainable way if management practices are complete. In fact, such valuable activities require appropriate action, and changes by the full range of societies and stakeholders involved in the conservation, production, and management as well as use of medicinal plants. Since an action on conservation and sustainable use of medicinal plants need involvement of various sectors and greater public support, it needs a continuous task of creating public awareness [45]. The review made by Megersa et al. [46] suggested that identifying serious threats to medicinal plants and how local people manage medicinal plants will help to understand the best conservation strategies.

## 4. Conclusions

A study on medicinal plant utilization in area revealed that the communities commonly use medicinal plants for maintaining their primary healthcare. The study resulted in documenting 70 medicinal plants species where Euphorbiaceae is the leading family with the highest proportion of medicinal plants. Most of (58) medicinal plants in the study area were harvested from wild. Shrubs were found to be the dominant growth form of medicinal plants used for preparation traditional remedies and followed by herbs. Roots were found to be the most frequently used plant parts for the preparation of traditional remedies. Traditional medicine preparation mostly involves a single plant and the method of preparation was mainly crushing followed by pounding. Depletion of indigenous knowledge among the people of the study area was serious due to disinterest of young generation to gain the knowledge. Oral based knowledge transfer, unavailability of the species, and influence of modern education were considered as the main factors. The main threat on medicinal plants in the study area arises from agricultural expansion. Utilization of root plant parts for traditional medicine preparation could also be considered as a threat. Although Berbere district was found to be rich in medicinal plant diversity, the effort to conserve the plants and associated indigenous knowledge was observed to be very poor. Thus, conservation of medicinal plants by local communities and responsible bodies is vital to avoid further loss. Moreover, phytochemical and pharmacological investigation is recommended with due consideration to frequently used medicinal plants.

## Figures and Tables

**Table 1:** List of medicinal plants used for human Ailments (Ha: habit, S: shrub, C: climber, T: tree, E: epiphyte, and H: herb, A/O-Afaan Oromo).

V. No	Family	Scientific name	Vernacular name (A/O)	Ha.	Ailments	Part used	Mode of preparation	Dosage	Route of administration
TT 34	Acanthaceae	*Barleria eranthemoides* R. Br. ex C. B. Cl.	Shabi	H	Heart burn	Root	The roots crushed and mixed with water and then given	Length of finger	Oral

TT 5	Acanthaceae	*Justicia schimperiana* T. Anderson	Dhumugaa	S	Bat urine	Leaves	The leaves are crushed; drink the juice and the residue put on the head for 7 days	5 leaves	Oral

TT 17	Aloaceae	*Aloe* sp.	Argiisa	S	Spleeno megally	Latex	The latex from leaf is taken with honey in the morning for 3 days	2-4 bottle stopper	Oral

TT 12	Anacardaceae	*Schinus molle* L.	Qundbarbare	T	Bile	Seeds	The seed powdered and boiled with tea then 1 cup is given for 3 mornings	3-5 seeds	Oral
					Spleeno megally	Leaves	The leaves are crushed and the filter is given for 5 days	5 leaflets	

TT 56	Anacardiaceae	*Rhus natalensis* (Bernh. ex Krauss) F. A. Barkley	Dabobessaa	S	Boils	Root	The root is crushed and ties on the infected area	1 local spoon	Dermal

TT 39	Apocynaceae	*Carissa spinarum* L.	Hagamsa	S	Wound	Root	Root bark is pounded & applied on wound for 3 days	Quarter of palm	Dermal
					Evil eye	Root	The smoke of pounded roots is inhaled	A palm of pounded root	

TT 36	Asteraceae	*Laggera crispata* (Vahl) F.N. Hepper & J.R.I. Wood	Rashadii	H	Dizziness	Leaves	The crushed leaf is tie on the head for 3 days	5-8 leaves	Dermal

TT 30	Asteraceae	*Xanthium strumarium* L.	Katerokantro	H	Dandruff	Leaves	The powdered root is painted on the head	5 leaves /treatment	Dermal

TT 37	Asteraceae	*Vernonia amygdalina* Del.	Dhebicha	S	Intestinal worm	Leaves	Drinking the decocted leaves with 1 cup of coffee for elders and half for children	3 -7 leaves	Oral
					Headache	Leaves	Crushed leaves are put on head for 3 days	10 leaves	Dermal

TT 11	Bignoniaceae	*Stereospermum kunthianum* Cham.	Wolgabis	T	Kidney	Bark	Drunk the juice from crushed bark & residue of the bark is steam bathed	5 spoon of crushed bark	Oral

TT 55	Boraginaceae	*Cordia africana* Lam.	Waddeessa	T	Itching	Root	The roots are powdered then painted at bed time for 5 days	1 hand palm	Dermal

TT 16	Boraginaceae	*Cynoglossum lanceolatum* Forrsk	Maxxannee	H	Fibril illness	Leaves	Juice of the leaf is taken.	Half to 1 stick	Oral
					Boils	Leaves	Fresh leaf is ground and then tie on	5 leaves	Dermal

TT 25	Boraginaceae	*Ehretia cymosa* Thonn.	Ulaagaa	S	Bleeding	Leaves	The squeezed leaves put on the cut area	1-5 leaves	Dermal
					Fibril illness	Leaves	The fluids from the leaves are taken through nose	2 leaves	Oral

TT 21	Canellaceae	*Warburgia ugandensis* Sprague	Befit	T	Boils	Stem	Pounded stem is concocted with butter and put on the infected part	1 sticken	Dermal
					Cough	Stem	The smoke of vascular part is inhaled to relief from cough	2-3 sticken	

TT 7	Capparidaceae	*Capparis sepiaria* L.	Gursama	S	Stomachache	Bark	The bark is pounded and drink with hot water.	3-5 spoon	Oral

TT 59	Capparidaceae	*Capparis tomentosa* Lam.	Lukkuu	S	Mental health problem	Root	The roots are powdered and then dry bathed for 3 days	1 palm of powdered root	Dermal

TT 18	Caricaceae	*Carica papaya* L.	Papaayee	S	Diarrhea	Seed	Seeds ground and boiled with coffee and taken with honey	15-20 seed	Oral
					Ascariasis				

TT 20	Cuccurbitaceae	*Cucurbita pepo* L.	Dabaqulaa	H	Gastritis	Leaves	The leaf cooked with *Brassica oleracea* then eaten	2-3 leaves	Oral

TT 65	Ebenaceae	*Euclea racemosa* L.	Mi'eesaa	S	Evil spirit& evil eye	Root	Drinking the Crushed & decocted root and inhaling the smoke	1 tea spoon	Oral
					Heartburn	Root	The root crushed and mixed with water and drink 1 cup of glass	1 tea spoon	

TT 31	Euphorbiaceae	*Bridelia scleroneura* Mul.Arg.	Abayi	T	Scabies	Seed	The seeds are crushed and applied on the affected area	5-8 seed	Dermal

TT 29	Euphorbiaceae	*Croton macrostachyus* Hochst. ex Del.	Bakkanisa	T	Hemorrhoids	Bark	Bark is crushed and cooked with meat then soup is taken	2 spoon	Oral
					Gonorrhea	Root	The root is decocted, and the mixture is taken.	3-6 spoon	
					Spleeno megally	Bark	Bark is crushed and mixed with water and then taken.	Quarter of palm	

TT 28	Euphorbiaceae	*Euphorbia* sp.	Guurii	H	Eczema	Leaves	The crushed and dried leaves are mixed with *citrus aurantifolia* and applied on the infected area	2-7 leaves	Dermal

TT 19	Euphorbiaceae	*Euphorbia heterophylla* L.	Annoo	H	Wound	Leaves	Leaves crushed and put on the wound for 3 days	2 spoon	Dermal

TT 40	Euphorbiaceae	*Euphorbia hirta* Millsp.	Qoricha aroo	H	Scabies	Root	The fresh root is crushed and painted on the body with butter for 5 mornings	1 stick of root	Dermal

TT 45	Euphorbiaceae	*Jatropha curcas* L.	Abatamuluk	S	Intestinal worm	Root	Root is crushed and drink with half cup of coffee	1 tea spoon	Oral
					Excess bile production	Seed	Powdered seed are eaten with honey	2 spoons	

TT 4	Euphorbiaceae	*Phyllanthus ovalifolius* A. Radcliffe-Smith	Gurbi adi	S	Scabies	Leaves	The leaves squeezed by hand and applied on the skin	5-17 leaves	Dermal

TT 69	Euphorbiaceae	*Ricinus communis* L.	Qobboo	S	Intestinal worm	Root and seed	Roots and seeds are crushed and drink with 1 cup of water	1-3 seeds and 2 sticken	Oral

TT 9	Euphorbiaceae	*Tragia cordata* Michx.	Lalesa	C	Urinary tract	Root	The dried roots crushed and boiled then drinks the decocted	Half to 1 full hand	Oral
					External parasite	Root	Powdered roots are applied to the affected cattle	5-9 local spoons	Dermal

TT 49	Fabaceae	*Acacia mellifera detinens* (Burch.) Brenan	Bilaal	S	Earache	Leaves	The leaf squeezed then droplet latex is applied to the ear	1 leaf	Auricular
					Eye disease	Leaves	The leaves are squeezed then the fluid is used as eye droplet	3-5 leaves	Opticular

TT 10	Fabaceae	*Acacia senegal* (L.) Willd.	Saphansa	T	Amebiasis	Latex from stem	Latex from the stem pounded and taken with honey	3 spoon	Oral
					Fire Wound	Latex	The powdered latex is applied on the wound	1-2 bean size solid latex	Dermal

TT 70	Fabaceae	*Calpurnia aurea* (Ait.) Benth.	Ceeka	S	Snake bite	Leaves	The decocted leaves are taken with Honey	20 leaflets	Oral
					Amebiasis	Root	The dried roots are crushed and boiled with leaf of coffee then 1 cup is given early in the morning	Half palm of hand	

TT 57	Fabaceae	*Rhynchosia ferruginea* A.Rich.	Udusalim baricha	S	Amebiasis	Root	The crushed roots are mixed with water and the mixture is taken	1-2 bottle stopper	Oral

TT 47	Fabaceae	*Rhynchosia malacotricha* Harms	Jidda dhiiga adii	S	Evil eye	Root	Dry bath of crushed root during bed time	1-3 spoon	Dermal

TT 13	Fabaceae	*Tamarindus indica* L.	Roka	T	Bile and intestinal worm	Fruit	The Fruit juice is taken with hot water in early morning before breakfast	Up to 10 fruits	Oral

TT 1	Iradiaceae	*Gladiolus schweinfurthii* (Baker) Goldblatt & M.P. de Vos	Mirge	H	Headache	Root	The pounded root is dry bathed for 1-3 days	1 tea spoon	Dermal

TT 60	Lamiaceae	*Clerodendrum myricoides* (Hochst.) R.Br. ex Vatke	Tiro	S	Earache	Root	Powdering the root and put small amount in the ear for 1hr and then wash	Half tea spoon	Auricular
					Headache	Root	The crushed root is put on the head with butter for 2 days	1 palm	Dermal

TT 15	Lamiaceae	*Ocimum lamiifolium* Hochst. ex Benth.	Urgo haree/ Damakase	S	Fibril illness	Leaves	The fluid from the squeezed leaves are taken	1-3 leaves depending on the age	Oral

TT 53	Lamiaceae	*Premna schimperi* Engl.	Urgeessaa	S	Mastitis	Root	The powdered roots are mixed with butter then painted on the breast of cows	5 hand palms	Dermal
					Boils	Leaves	crushed & pounded leaves are tied on the infected body	3 spoons	Dermal

TT 66	Loranthaceae	*Erianthemum dregei* (Eckl. & Zeyh.) van Tiegh.	Mankero mi'esa	E	Hyper menorrhea	Leaves	The leaves are crushed, and the juice is taken after 3 days of blood flow	5 leaves	Oral

TT 51	Malvaceae	*Sida rhombifolia* L.	Gurbi	S	Epitaks/nose bleeding	Leaves	Leaf is squeezed and the liquid taken through the nose during bleeding	2 leaves	Nasal

TT 38	Menispermaceae	*Stephania abyssinica* (Dill. & A. Rich.) Walp.	Baltokkee	Cl	stomachache	Root	The root is chewed, and the juice is swallowed	forefinger size	Oral

TT 9	Moringaceae	*Moringa stenopetala* (E. G. Baker) Cufod.	Shiferawu	T	Asthma	Root	The smoke of pounded root is inhaled at bed time for 3 days	Half palm	Oral

TT 62	Olacaceae	*Ximenia americana* L.	Hudhaa	S	Stomachache and tonsillitis	Root	The roots are crushed, pounded and then 1 cup is taken for 3 days	2 spoons	Oral
					Wound	Fruit	The oil from the fruit kernel is applied to flesh wounds to prevent infections	1 bottle stopper	Dermal

TT 42	Oleaceae	*Olea europaea* subsp. *cuspidata* (Wall.G.Don)Cif.	Ejersa	T	Bone TB	Root	The extracted oil from the roots put on the affected site	Half of tea spoon	Dermal
					Wound				

TT 43	Plumbaginaceae	*Plumbago zeylanica* L.	Dhigaji	H	Gonorrhea	Root	The crushed root is cooked with meat and then eaten	2 sticken	Oral
					Hemorrhoids	Root	The pounded roots are mixed with honey and then put in the anus	Half palm	Dermal
					Toothache	Root	Small amount of crushed root put on the infected teeth	1 sticken	

TT 14	Polygalaceae	*Polygala erioptera* DC.	Harmal adii	H	Heart disease	Root	Pounding the root and chewing then drinking	1-3 sticken	Oral

TT 58	Ranunculaceae	*Clematis simensis* Fresen.	Sariti/ fitii	Cl	Wound	Leaves	The crushed leaves are mixed with citrus lemon and then put on the wound	2 leaves	Dermal
					Stomachache	Root	The root pounded then taken with coffee for 3 days	The size of Fore finger	Oral

TT 41	Rosaceae	*Prunus africana* (Hook.f.) Kalkman	Muka guracha	T	Wound	Bark	Crushed bark applies on the wounded area	Quarter palm	Dermal

TT 35	Rubiaceae	*Coffea arabica* L.	Buna	S	Scabies	Seed	The roosted seeds are powdered and applied on the infected area	5-7 seeds	Dermal

TT 23	Rutaceae	*Citrus aurantium* L.	Harboo	S	Dermatophyte	Fruit	Fruit juice is used as cream ointment on head	3 fruits	Dermal

TT 64	Rutaceae	*Citrus sinensis* (L.) Engl.	Burtukana	S	Stomach infection	Fruit	The juice is mixed with the fruits of *Psidium guajava* and taken early	1-3 fruits	Oral
					Wound	Bark	The dried bark is crushed and mixed with butter then creamed on the wound after washing the infected part	Half palm	Dermal

TT 54	Rutaceae	*Ruta chalepensis* L.	Chelatama	S	Stomachache	Leaves	The leaves are chewed and the juice swallowed	5 -10 leaf	Oral
					Cough	Fruit	The fruits are boiled with tea and taken in the morning and night for 2 days	Up to 6 fruits	

TT 24	Sapindaceae	*Dodonaea angustifolia* (L. fil.) J.G.West	Itachaa	S	Toothache	Root	Brushing the teeth	1 stricken	Oral
					Wound	Root	Squeezed root is smeared on the wound	Half spoon	Dermal

TT 48	Sapotaceae	*Mimusops kummel* Bruce ex A. DC.	Qolati	T	Lung cancer	Root	The roots are grounded, and small quantity taken with water	1 tea spoon	Oral
						Fruit	The dried fruits are pounded and dissolved in water and given	3 fruits	

TT 33	Solanaceae	*Datura stramonium* L.	Manjii	H	Depression	Seed	The seed is grounded and smoked or mixed with butter and put on head	15 seeds	Dermal

TT 44	Solanaceae	*Nicotiana tabacum* L.	Tamboo	H	Snake bite	Leaf	The leaf is chewed and the juice swallowed	1 leaf	Oral

TT 52	Solanaceae	*Solanecio angulatus* (Vahl) C.Jeffery	Jiniras	H	Evil eye	Root	The powdered root is dry bathed at night for 3 days	1 spoon	Oral

TT 67	Solanaceae	*Solanum americanum* Mill.	Mujulo	H	Intestinal worm	Leaves	The dried leaf is crushed	7 leaves	Oral

TT 68	Solanceae	*Solanum incanum* L.	Hiddii	S	Emergency	Root	Chewing the root	Size of 1 sticken	Oral
					Nose bleeding	Leaf	Juice of leaf is applied to nose	1 leaf	Nasal
					Pain during menstruation	Root	Dried roots are chewed and juice intake to stomach	Half sticken	Oral
					Amebiasis	Root	Pounded roots are concocted with water and then taken	2 sticken	Oral

TT 63	Solanaceae	*Withania somnifera* (L.) Dun.	Unzo/ Gizawa	H	Evil eye	Root	Drinking the decocted roots and the residue is dry bathed	Half palm	Oral

TT 32	Tiliaceae	*Grewia* sp.	Gurbi dima	S	External parasite	Leaves	The leaves are squeezed and rubbed on the body	5-15 leaves	Dermal

TT 46	Tiliaceae	*Grewia villosa* Willd.	Jidda dhiga dima	S	Headache	Root	Pounding the roots and dry bath during bed time	1 and half palm	Oral

TT 2	Trymalaceae	*Gnidia stenophylla* Gilg.	Katarichaa	H	Intestinal worm	Root	The dried roots are crushed and mixed with water then taken for 2 days	1 tea spoon	Oral
					Amebiasis	Root	The powdered roots are taken with coffee in morning	1-2 spoon	
					Liver disease	Root	The decoction of root is taken with goat milk	1 tea spoon	

TT 61	Ulmaceae	*Celtis africana* Burm. fil.	Mataqoma	T	Boils	Leaves	The leaves squeezed and tie on the swelled part	5 leaves	Dermal

TT 7	Verbenaceae	*Lippia adoensis* Hochst. ex Walp.	Kusayee	S	Fibril illness	Leaves	The leaves squeezed and the filter is given through the nose and drink	2 leaves	Oral

TT 50	Vitaceae	*Cayratia ibuensis* (Hook.f.) Suess.	Udusalim rumiyi	S	Liver disease	Root	The roots crushed and pounded then boiled and drink 2-3 cup of coffee in the morning	5-7 of tea spoon	Oral

TT 27	Vitaceae	*Cissus rotundifolia* (Forssk.) Vahl	Burke	C	Swelling	Root	The crushed roots are tie on the swelled part	1-5 full hand	Dermal
					Wound	Root	Roots are pounded, mixed with water, rubbed and applied as ointment on wound	2 hand palms	

TT 22	Vitaceae	*Cyphostemma* sp.	Laaluu	C	Wound	Root	Root is pounded, mixed with water, rubbed and applied as ointment on wound	2 bottle stopper	Dermal

TT 26	Zingiberaceae	*Zingiber officinale* Roscoe	Zinjibilaa	H	Cough, common cold & tonsillitis	Root	The roots crushed and boiled with tea and then taken	2-5 medium root	Oral

**Table 2:** Preference ranking of medicinal plants used for treating stomachache.

Medicinal plants used	Respondents (A-J)										Total	Rank
	A	B	C	D	E	F	G	H	I	J		
*Solanum incanum *	5	4	5	5	4	5	4	4	5	5	46	2
*Stephania abyssinica*	5	5	5	5	5	5	5	5	5	5	50	1
*Carissa spinarum*	4	3	4	5	4	3	2	3	5	4	37	4
*Calpurnia aurea *	5	5	3	3	5	4	5	5	4	3	42	3
*Croton macrostachyus*	4	3	2	3	5	4	3	4	3	5	36	5

**Table 3:** Paired comparison of medicinal plants used to treat wound (As: *Acacia senegal*, C. sp: *Cyphostemma* sp., Eh: *Euphorbia heterophylla*, Oe: *Olea europaea* subsp *Cuspidata*, and Pa: *Prunus africana*).

Plant species	*Acacia Senegal*	*Clematis simensis*	*Cyphostemma *sp.	*Euphorbia heterophylla*	*Olea europaea *subsp* Cuspidata*	*Prunus africana*
*Acacia Senegal*						
*Clematis simensis*	As					
*Cyphostemma sp.*	As	Csp				
*Euphorbia heterophylla*	Eh	Eh	Eh			
*Olea europaea *subsp* Cuspidata*	Oe	Oe	Oe	Oe		
*Prunus africana *	Pa	Pa	Pa	Pa	Oe	
Frequency	2	0	1	3	5	4
Rank	4	6	5	3	1	2

**Table 4:** Informant consensus factor by categories of diseases in the study area.

Disease categories	No. of species	Use citation	ICF
Asthma, cough, earache, epitaks, headache, Tonsillitis and Toothache	6	19	0.72
Epilepsis, evil spirit, evil eye, doziness	13	47	0.74
Bat urine, bile problem, splenomegaly, flunk pain and urinary retention	17	55	0.70
Gonorrhea and syphilis	3	39	0.95
Amoebiasis, appetite loss, diarrhea, gastritis, intestinal worm, stomachache and vomiting	8	37	0.80
Emergency and fibril illness	5	15	0.71
Rabies and Snake bite	2	11	0.9
Boils, dandruff, eczema, hemorrhoids, scabies, taenia resicolors and wound	11	33	0.69

**Table 5:** Direct matrix ranking of six plant species by four participants based on six use criteria.

Use categories	Medicinal plants																							
	*Warburgia ugandensis*				*Olea europaea *subsp* cuspidata*				*Acacia senegal*				*Prunus africana*				*Carissa spinarum*				*Croton macrostachyus*			
	Participants (1-4)				Participants				Participants				Participants				Participants				Participants			
	**1**	**2**	**3**	**4**	**1**	**2**	**3**	**4**	**1**	**2**	**3**	**4**	**1**	**2**	**3**	**4**	**1**	**2**	**3**	**4**	**1**	**2**	**3**	**4**
Charcoal	4	3	4	3	1	2	0	2	3	4	2	1	4	3	2	1	1	2	2	1	5	4	5	4
Construction	5	5	5	5	5	4	2	4	1	2	2	2	4	4	2	1	3	2	3	3	4	3	3	3
Fence	3	2	1	2	2	3	1	3	5	5	4	5	3	3	2	2	5	4	5	5	5	4	5	4
Fire wood	5	5	5	5	5	5	5	5	5	4	5	5	5	5	4	4	4	5	5	5	5	5	4	5
Furniture	1	2	3	2	3	4	3	2	2	2	2	1	2	1	1	0	1	1	0	1	2	2	3	2
Medicinal	5	4	5	5	5	5	5	4	4	4	5	4	4	5	4	4	5	5	5	5	5	5	5	5
Indi. Total	23	21	25	22	21	23	16	20	20	21	20	18	22	21	15	12	19	19	20	20	26	23	25	23
Grand total	91				80				79				70				78				97			
Rank	2				3				4				6				5				1			

## Data Availability

The data used in this study is available from the corresponding author upon request.
